# Herpes simplex virus-infected squamous cell carcinoma: a case report

**DOI:** 10.1186/s12879-021-06995-8

**Published:** 2022-01-04

**Authors:** Sarah H. Brown, Vanessa A. R. States, Abaseen K. Afghan, Gowri Satyanarayana

**Affiliations:** 1 Vanderbilt University School of Medicine, 1161 21st Ave S #D3300, Nashville, TN 37232 USA; 2 Department of Pathology, Microbiology, and Immunology, Vanderbilt University Medical Center (VUMC), Nashville, TN USA; 3 Department of Medicine, Division of Infectious Diseases, VUMC, Nashville, TN USA

**Keywords:** Herpes simplex virus, Squamous cell carcinoma, Stem cell transplant, Case report

## Abstract

**Background:**

Herpes simplex virus (HSV)-1 is a highly prevalent, non-oncogenic virus that has higher morbidity in immunocompromised hosts. Its most common clinical manifestation is superficial ulceration of the integument or mucus membranes.

**Case presentation:**

A 65-year-old woman with a history of acute myelogenous leukemia treated with allogenic peripheral blood stem cell transplant presented for resection of an ulcerated buccal squamous cell carcinoma. We report a case of HSV-1-infected malignant cells discovered on histopathological examination of the carcinoma specimen ultimately treated with valacyclovir.

**Conclusions:**

HSV-1 is not considered an oncogenic virus itself but may increase risk of malignant progression. Cancer cells are vulnerable to superimposed viral infections, including HSV-1, which likely led to the findings in this case.

## Background

Herpes simplex virus (HSV)-1 is a common virus with seroprevalence of 54% in the United States and higher seroprevalence of 80–90% reported in some adult populations [1, 2]. One population that is particularly at risk for severe or unusual HSV-1 infection is transplant recipients. We report a case of a hematopoietic stem cell transplant recipient whose resected left buccal squamous cell carcinoma (SCC) showed HSV-1 infection of malignant cells on histologic examination of the surgical specimen, a rare finding.

## Case presentation

**Fig. 1:**
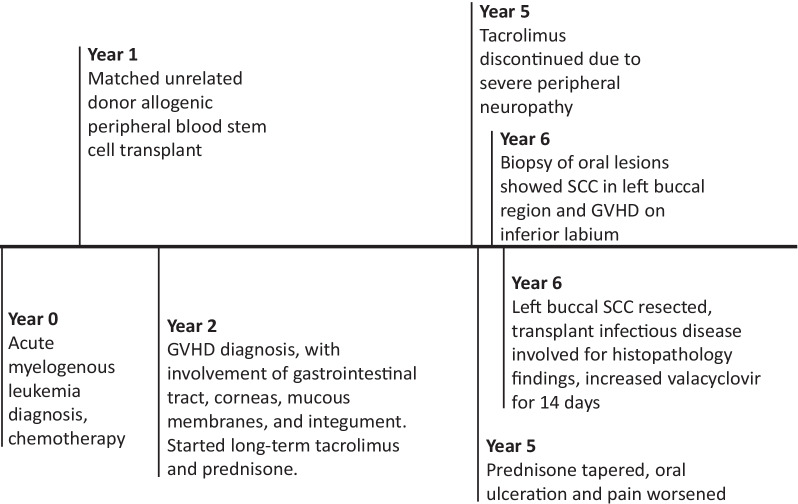
Timeline. The patient’s course included a long history of graft versus host disease (GVHD) requiring immunosuppression. When the peripheral neuropathy from tacrolimus became too severe, the tacrolimus was stopped and prednisone tapered. Around the same time, the patient experienced worsening oral ulceration and pain. Initial biopsy of her oral lesions showed squamous cell carcinoma (SCC) in the left buccal tissue and GVHD in the inferior labium. It was not until full resection of the buccal SCC that herpes simplex virus-1 was identified on microscopic examination of the surgical specimen. *GVHD* graft versus host disease. *SCC* squamous cell carcinoma

**Fig. 2:**
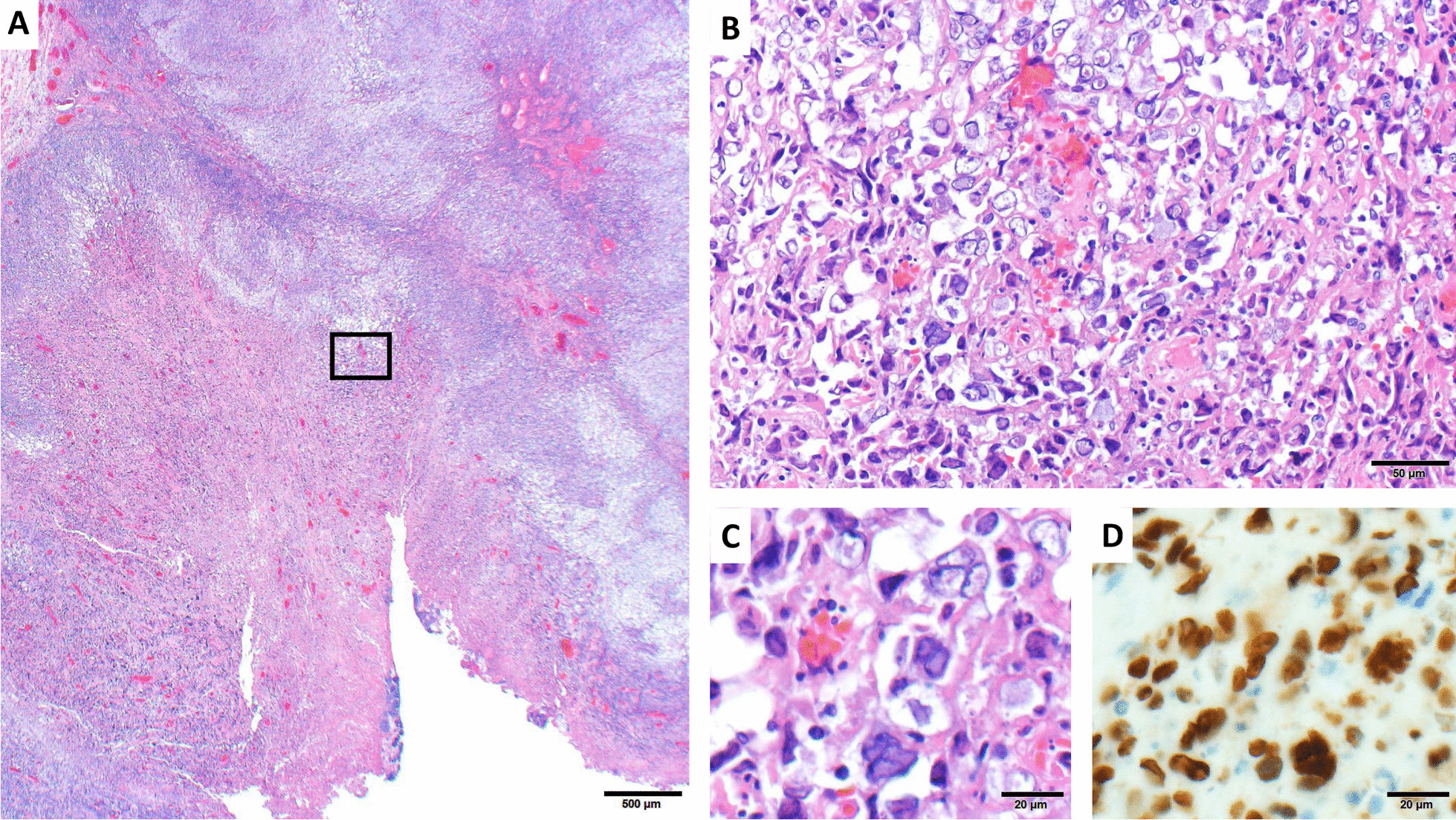
Hematoxylin and eosin (H&E)-stained photomicrographs of the left buccal tumor. Low-power examination (**A**, 20X magnification) shows invasive moderately differentiated squamous cell carcinoma with superficial ulceration. Higher-power magnification (**B**, 200X magnification and **C**, 400X) reveals numerous multinucleated malignant cells with glassy eosinophilic viral inclusions, which are strongly positive for HSV by immunohistochemistry (**D**, 400X magnification)

A 65 year-old woman with a history of acute myelogenous leukemia treated two years prior with allogenic peripheral blood stem cell transplant from an HLA-matched unrelated donor presented for left buccal squamous cell carcinoma resection. Her transplant course was complicated by severe graft versus host disease (GVHD) affecting multiple organ systems including the oral mucosa that required long-term immunosuppression with tacrolimus and prednisone. She discontinued tacrolimus five months prior due to peripheral neuropathy and was prescribed a prednisone taper, taking 10 mg daily at the time of presentation (Fig. 1). Reduction in immunosuppression led to increased painful ulceration of her oral mucosa. Initial diagnostic biopsy of two oral lesions showed inferior labial “mild dysplasia and lichenoid inflammation” consistent with GVHD as well as left buccal SCC. One month later, the two regions were excised. Histopathologic evaluation with Olympus BX41 microscope and image capture with Olympus cellSens software (72 dpi with no downstream processing) of the left buccal resection specimen showed multinucleated tumor cells with glassy eosinophilic nuclear inclusions (Fig. 2A–C). An HSV I and II immunohistochemical stain using mouse monoclonal antibodies that react with viral envelope glycoproteins and core proteins (clone: 10A3/BSB-116, isotype: IgG2a) demonstrated strong nuclear staining, confirming superimposed HSV infection (Fig. 2D). The lower lip specimen showed invasive, well-differentiated squamous cell carcinoma without evidence of superimposed viral infection.

Upon clinical exam, the patient had crusted herpetic lesions on her inner inferior labium, a subtle finding in the context of post-surgical trauma from the SCC resection and facial reconstruction. She endorsed having oral ulcers prior to surgery, although it was difficult to distinguish the ulcers clinically as caused by HSV, GVHD, or focal SCC. On the initial biopsies described previously, only the latter two were identified.

HSV-1 DNA was detected in a sample swabbed from the base of the labial ulcer by real-time PCR. The serum anti-HSV Ab titer was not collected. She was treated with a 14-day course of valacyclovir 1000 mg three times daily without complication and recommended to start HSV prophylaxis after treatment.

## Discussion and conclusions

Immunodeficient patients, including post-transplant patients, are at higher risk of morbidity and mortality from HSV infections [2]. Recipient HSV serology is commonly obtained prior to hematopoietic stem cell transplant [3]; however, possibly due to being many years post-transplant, this patient did not undergo evaluation for HSV until signs of viral infection were seen in the SCC pathology. This infection could have been a primary infection, in which HSV-1 in saliva initially replicated in epithelial cells causing ulceration or replicated in already-damaged carcinoma cells. The infection may have also been secondary, in which the virus replicated after a latent period. Latency occurs when HSV-1 infects sensory neuron termini—in this case, the trigeminal ganglion—and travels retrograde to the cell body. Secondary infection occurs when the virus reactivates and travels anterograde to the skin or mucosa, replicates in the epithelium, and causes active infection [4]. In this case, serologic testing for HSV-1 IgG and IgM was not performed because it would not have changed clinical management. HSV Ab testing can also be difficult to interpret. IgG positivity can be present in both chronic infections and acute disease, while IgM is negative in 50% of initial infections and can recur in secondary infections [5].

Although HSV-1 itself is not an independently oncogenic virus, it can interact with viruses linked to cancer development including Epstein Barr virus (EBV) and human papillomavirus (HPV). HPV definitively causes cervical cancer and is likely linked to oral SCC, while EBV causes Burkitt lymphoma or nasopharyngeal carcinoma and is possibly, but not definitively, linked to oral SCC. HSV-1, on the other hand, has been identified in cancer tissues and is shown to disrupt cellular DNA damage repair and enhance preexisting oncogenes [6–8]. A single case report describes a presumably immunocompetent patient with a surgical vulvar SCC specimen found to have viral inclusions in the malignant cells with positive immunohistochemistry for HSV [9]. In one study examining the prevalence of HSV, HPV, and EBV in oral SCC samples, 15% were positive for HSV and co-infection was detected [8]. Studies suggest that HSV-1 and concurrent tobacco or chemical carcinogen exposure may promote oral SCC development, presumably through increased oncogene expression facilitated by HSV-1, but our patient never used tobacco and rarely consumed alcohol. Co-infection of HSV with EBV or HPV may promote oral SCC development through a similar mechanism and could elevate risk of progression to malignancy [10]. Additional immunohistochemistry for EBV and HPV were not performed on the surgical specimen. The patient did have EBV seropositivity with a history of intermittent low level reactivation and high risk-type HPV positivity, as well as numerous compounding risk factors for SCC including personal history of SCC, hematopoietic stem cell transplant, and chronic GVHD requiring prolonged immunosuppression which can increase susceptibility to both viral infection and malignant transformation. [7, 11, 12].

HSV-1 infection of the SCC was likely to be incidental since there was no evidence of cellular changes consistent with superimposed infection on the initial biopsy of SCC. HSV-1 and other viruses can replicate preferentially in oncogenic tissue because cancer cells often have disrupted anti-viral defense pathways. For example, Talimogene laherparepvec (TVEC) is a genetically-modified oncolytic HSV-1 approved to treat advanced melanoma that, like the wildtype virus, propagates in tumor tissues by exploiting disrupted anti-viral pathways, namely protein kinase R and type I interferon [13].

Valacyclovir treatment can reduce pain and promote healing of herpes labialis if the treatment is initiated rapidly. Although the timeline of the infection was unclear, the patient was beyond the prodromal phase since she had active oral ulceration, but may have been within 48 h of initial infection, the preferred treatment initiation window [14]. HSV infection of the SCC identified incidentally on histology ultimately did not cause significant morbidity to this patient.

## Data Availability

Not applicable.

## Abbreviations

GVHD: Graft-versus-host disease
HSV-1: Herpes simplex virus-1
SCC: Squamous cell carcinoma
